# Muscovite based polyaniline nanocomposite as effective adsorbent for removal of Cd^2+^ and Pb^2+^ ions from liquid waste

**DOI:** 10.1038/s41598-025-99686-2

**Published:** 2025-06-20

**Authors:** M. Nageeb Rashed, A. S. A. Arifien, F. A. El-Dowy

**Affiliations:** Chemistry Department, Faculty of Science, Aswan University, Aswan, Egypt

**Keywords:** Polyaniline, Muscovite, Nanocomposite, Adsorption, Lead, Cadmium, Environmental sciences, Chemistry, Nanoscience and technology

## Abstract

Heavy metals in wastewater represent a main source of environmental contamination for the ecosystem and aquatic system. Herein, the in situ polymerization method was used to prepare a novel nanocomposite polyaniline/muscovite (PANI/Msc) using ammonium persulphate as an oxidizing agent and HCl as a catalyst. The combination of muscovite with polyaniline (PANI) creates a hybrid material that leverages the strengths of both components. PANI/Msc nanocomposite was characterized using XRD, TEM, FT-IR, SEM, and surface area analyzer. Crystalline size of the prepared nanocomposite found in the range 15.6 – 45 nm , while its surface area was 208.6 m^2^/g. The resulting nanocomposite was used for Cd^2+^ and Pb^2+^ adsorption from their solution. Up to 75.6 and 72.6% of Cd^2+^ and Pb^2+^, respectively, were removed in the optimized conditions of metal concentration 75 ppm, pH 6 and 7 for Pb^2+^ and Cd^2+^, adsorbent dose 0.1 g, 25 °C solution temperature, and 60 min contact time. Kinetic and adsorption studies clearly demonstrated that the results of the adsorption process followed the pseudo-second order (q_e_ 32.8 and 33.1 mg g^−1^) and Langmuir models (Q_0_ 36.1 and 61 mg g^−1^) for Cd^2+^ and Pb^2+^, respectively. The thermodynamic indicated favorable, spontaneous and exothermic process. Electrostatic interaction and ion exchange mechanisms were responsible for the adsorption of Cd^2+^ and Pb^2+^ by PANI/Msc as demonstrated by FTIR spectroscopy and pH studies. This study ended with the cost- effective preparation of PANI/Msc nanocomposite that offers a promising solution for the removal of heavy metals from contaminated water source. In addition, the capability study regarding Pb^2+^ and Cd^2+^ion adsorption over the PANI/Msc nanocomposite clearly revealed that our method is suitable for large- scale application.

## Introduction

Wastewater containing heavy metal pollutants, when discharged to surface water, causes water pollution and human health. As a result, it is now a global challenge to have access to clean water for drinking and irrigation. It is challenging to remove and recover metal ions from aqueous effluent because of their diverse physical and chemical properties, making it difficult to completely remove and recover them. Pollutants containing heavy metals are introduced into water systems by industrial, mining, and other sources^1^.

There have been a number of technologies reported for the removal of heavy metals from contaminated water, including complexation, precipitation, membrane filtration, flotation, oxidation, solvent extraction, adsorption, photocatalysis, ion exchange electrodialysis, reverse osmosis, electrochemical and electrocatalysis methods^2–7^. However, the adsorption method is thought to be one of the most effective, practical, and easy to apply. It is considered cost-effective technology for treating wastewater^8–10^.

Different adsorbents have produced effective treatment of wastewater, which include zeolites^11^, activated carbons^12^, natural clay minerals^4^, sludge materials, agricultural waste, biochar, polymeric resins, and industrial waste^10^.

Recent advancements and methodologies of pollutant removal have been studied. Advanced conditioning techniques, such as those explored by Deng, et al.^13^, demonstrate enhanced solid–liquid separation efficiencies, which may inform the design of adsorption systems. Recent studies by Wang, et al.^14^ on the removal of organic and inorganic contaminants underscore the potential of hybrid adsorption-oxidation systems in water treatment. Li, et al.^15^ highlighted innovative oxidative coupling strategies, providing insights into improving adsorption efficiency for pollutant removal. The simultaneous removal of SO₂ and NO as reported by Li, et al.^16^ exemplifies the integration of multiple functionalities in environmental remediation technologies. Stress-mediated modifications, as studied by Xie, et al.^17^, show promise in enhancing the surface reactivity of adsorptive materials. Ali Alshehri and Pugazhendhi^18^ emphasized the role of biochar and composite materials in achieving efficient contaminant removal, applicable to the current study’s system. Green recycling processes, like those explored by Yu, et al.^19^, provide a sustainable perspective on material recovery and reuse, potentially aligning with the regenerative aspects of adsorbents.

Because of their affordability and practical uses as adsorbent materials, clay minerals are widely employed. However, the utilization of natural clay minerals is restricted due to their small surface area and the absence of standardized regeneration and recovery processes in aqueous systems.

Contrarily, polymers have demonstrated the ability to overcome clay minerals’ limitations^20,21^. Clay-polymer nanocomposites (CPNs) have received much interest from researchers to be used as adsorbents^22^. Due to their diverse uses and significant benefits in a variety of industries, including water purification, CPNs synthesis is growing in popularity. A compound called polymer nanocomposite (PCN) has many more desirable properties than its component polymers^23^.

Because of its low cost of raw materials, high electrical conductivity, easy synthesis, environmental stability, unique conductivity properties, high stability, redox properties, relatively high porosity, and high surface area, polyaniline PANI has attracted a lot of research interest among conductive polymers^24^.

Utilising simple synthesis techniques, innovative nanocomposite adsorbents made of polyaniline and another adsorbent with superior adsorption characteristics, strong regeneration ability, and selectivity have been created. These nanoparticles are frequently employed in water treatment; because of their electrical, mechanical, improved conductive, and adsorbing properties^25,26^.

Solvent intercalation, melt compounding, and in situ, polymerization are techniques used to create clay-polymer nanocomposites. In situ polymerization and solvent intercalation both enable polymer chains to penetrate silicate clays’ galleries^27^.

By using in-situ polymerization, Kalotra and Mehta^22^ prepared nanocomposites (polyaniline/clay), and used them to remove the acid green dye from wastewater. Liu, et al.^28^ prepared polyaniline/vermiculite nanocomposite via in situ polymerization of aniline. Vijayakumar, et al.^29^ synthesized polyaniline/smectite nanocomposite by heating aniline and ammonium persulfate in the presence of clay at 0–5 °C for 12 h. By utilizing ammonium persulfate as an oxidant in a solid process, Bekri-Abbes and Srasra^30^ produced the polyaniline (PAn) and polyaniline/montmorillonite (Mt) nanocomposite.

In situ, polymerization was used by Kalotra and Mehta^22^ to develop nanocomposites of polyaniline and polyaniline/montmorillonite clay. Tang, et al.^31^ prepared nanocomposites (polyaniline/vermiculite) by in situ polymerization. Piri, et al.^32^ prepared polyaniline/clay and used it in water treatment.

Muscovite is a member of the mica family of clay minerals. Natural 2M1 Muscovite (Silicate mineral with a 2:1 phyllosilicate ratio) has the chemical formula KAl_2_(Si_3_AlO_10_)(OH)_2_ with layered silicate mineral created when biotite and iron-bearing phlogopite micas weather together. The mica group minerals consist of two tetrahedral SiO_4_ sheets and an octahedral Al(O/OH)_6_sheet^33^.

Muscovite advantages over other natural adsorbents are low cost as an adsorbent, high availability, and ease of handling^34^. Rashed, et al.^35^ used chemically activated Muscovite as an adsorbent of heavy metals.

The interlayer area should ideally be occupied while creating layered silicate nanocomposite materials. So, the ability of layered silicates to exchange charge to cations has led to substantial research and development^36^.

Rashed, et al.^37^ synthesize Muscovite nanoparticles using intercalation agents (PA- TTAB-DTAB- DTPA- PN) with its application in the removal of Pb^2^ and Cd^2+^ from wastewater. Using K-feldspar, Yuan, et al.^38^ prepared nano muscovite. Chen, et al.^39^ prepared exfoliated Muscovite/poly (2, 3-dimethylaniline) nanocomposite. Silica-based nanomaterials have been employed for metal adsorption, because of their extremely adsorptive surface area and pore size.

Preparation of Muscovite nanocomposites was via intercalation with ion exchange between inorganic and organic phases^40^. Ismail, et al.^41^ synthesized nanomuscovite using cetyltrimethylammonium bromide and alkaline salt.

Kaolinite/muscovite composite was prepared by activation agents (NaOH and H_2_SO_4_) and applied it for Pb^2+^adsorption^42^. As a protective coating for mild steel surfaces, Senthilnathan, et al.^43^ created muscovite polyaniline nanocomposites with superior anti-corrosive properties.

This study aims to prepare polyaniline Muscovite nanocomposite as a new adsorbent for the adsorption of cadmium and lead from liquid waste, focusing on the optimum condition for the maximum adsorption of cadmium and lead from its solution.

The novelty of this work focuses on increasing the surface area and electrostatic properties of nanomuscovite, enabling the optimization of adsorption properties for specific heavy metals, by simply prepared PANI-Muscovite nanocomposite via a combination of polyaniline (PANI) and nanomuscovite which novel and has not been extensively explored.

## Material and methods

### Sample collection

Bulk Muscovite samples of 10 kg were gathered in Egypt’s southeast desert. Using an agate mortar, the samples were broken up and processed into powder. The powdered muscovite samples were soaked in deionized water for a day, the silica particles from the samples of Muscovite were eliminated, and the solution was then filtered before being dried at 60 °C for 24 h and being sieved to a 63 μm mesh size.

### Reagents and chemicals

High analytical—grade chemicals and reagents were used: cadmium and lead standard solutions (1000 ppm) were perched from BDH, UK. Ammonium persulphate (NH_4_)_2_S_2_O_8_, Alfa Aesar DTAB (1-Dodecyl) trimethyl ammonium bromide (CH_3_(CH_2_)_11_N(CH_3_)_3_Br, and aniline (C_6_H_5_NH_2_) were of analytical grade supplied by BHD Chemical Ltd., UK. Aniline was purified before its use as the precursor in the polymerization reaction. Ammonium persulphate was selected as the oxidizing agent for polyaniline synthesis, while DTAB used as an intercalate agent to prepare the nanomuscovite.

### Preparation of metal standard solutions

Working standard Pb^2+^ and Cd^2+^ solutions were prepared by diluting the stock one (1000 ppm).

### Instruments and tools

A flame atomic absorption spectrophotometer Varian GBC 932 AA was used in the quantitative analysis of metal ion concentration.

### Characterization techniques

Morphological analysis of polyaniline Muscovite nanocomposite [PANI/Msc] was performed. Power X-ray diffraction patterns (XRD) were performed for the PANI/Msc nanocomposite on a Bruker D advance XRD meter between angle 2θ = 5–60° at 40 kV using X-rays diffraction patterns (Brukeraxs D8, Germany). Scanning electron microscopy (SEM–EDX, JEOL, JSM-5500 LV electron microscopy) technique was used to morphological analyses and characterization of the size and shape of the resulted nanocomposite. Transmission Electron Microscopy (TEM) Investigation was performed using TEM, JEOL-JEM-1230, Tokyo, Japan. The chemical bond characteristic of the PANI/Msc nanocomposite was acquired by Fourier transform infrared (FTIR) spectroscopy (FTIR, JASCO 3600, Tokyo, Japan using KBr pellets in the 400–4000 cm^−1^ frequency range. Surface area BET techniques was employed to determine the surface area, pore volume and pore size of the prepared adsorbent by Quatachrome Instruments (NOVA 2000 series, UK).

The integration of XRD, SEM–EDX, TEM, FTIR, and surface area BET techniques in this study allowed for a comprehensive understanding of the structural, and chemical properties of the synthesized samples, facilitating a nuanced analysis of their composition and potential applications.

### Preparation of polyaniline muscovite nanocomposite [PANI/Msc]

Our previously published research showed that DTAB (1-Dodecyl trimethyl ammonium bromide) was used to prepare the nanomuscovite as published by Rashed, et al.^37^. The PANI/Msc nanocomposite was prepared with aniline polymerization on the exfoliated nanomuscovite. In a 50 mL solution of 1 M HCl, several ratios of nanomuscovite and aniline were added (Table 1). After being sonicated for one hour, the mixture received 5 g of ammonium persulphate (APS) dissolved in 30 mL of 1 M HCl solution, followed by stirring for 7 h at 25 °C. Following filtration, the precipitates were twice washed with distilled water and vacuum-dried for 24 h at 25 °C. The resulted polyaniline Muscovite nanocomposites were labelled as PANI/Msc1, PANI/Msc2, and PANI/Msc3 (Table 1).

**Table 1:** Ratio of aniline and NanoMuscovite.

Aniline (ml)	Nanomuscovite (g)	Ammonium persulphate (APS)	Weight PANI/Msc (%) Nanomuscovite/polyaniline
0.5	2.2	1.25 g in 7.5 ml HCl	50
1	2.2	2.5 g in 15 ml HCl	25
0.5	4.4	1.25 g in7.5 ml HCl	89

### Adsorption properties of polymer muscovite nanocomposite [PANI/Msc]

The adsorbed amount of each metal (qe) and removal percentage (R%) for all conducted experiments was determined by these equations.

1 $${\text{qe}}\;{\text{ = (C}}_{{\text{o}}} {\text{ - C}}_{{\text{e}}} {)}{{\text{V}} \mathord{\left/ {\vphantom {{\text{V}} {\text{m}}}} \right. \kern-0pt} {\text{m}}}$$

2 $${\text{R\% }}\;{ = }{{{\text{(C}}_{{\text{o}}} {\text{ - C}}_{{\text{e}}} {)}} \mathord{\left/ {\vphantom {{{\text{(C}}_{{\text{o}}} {\text{ - C}}_{{\text{e}}} {)}} {{\text{C}}_{{\text{o}}} {\kern 1pt} {\text{X}}{\kern 1pt} {\kern 1pt} 100}}} \right. \kern-0pt} {{\text{C}}_{{\text{o}}} {\kern 1pt} {\text{X}}{\kern 1pt} {\kern 1pt} 100}}$$

where C_o_ and C_e_ are initial and equilibrium concentrations of metal in (mg L^−1^), respectively, while V and m are the volume of the aqueous metal solution (L) and dosage of adsorbent (g), respectively.

All the experiments were performed in triplicates, and the average values were used for experimental data evaluation.

#### pH Effect

The pH level of a solution significantly affects adsorption studies due to its influence on adsorbate solubility and the concentration of metals in adsorbent functional groups. To an aliquot of 25 mL of metal solution concentration (50 ppm Pb, Cd) in an Erlenmeyer flask (50 mL) was added to 0.1 g of PANI/Msc nanocomposite, and the solution mixture was shaken on a shaker at a different pH (2, 4, 5, 6, 7, 8, and 10) for 1 h, and filtered. In the filtrate, lead and cadmium were measured by atomic absorption spectrophotometer.

#### Adsorbent doses

Adsorbent dosage is known to have a great impact on the overall adsorption process. In order to study the effects of different PANI/Msc doses on the adsorption efficiency of Pb and Cd ions, different doses of PANI/Msc (0.05, 0.1, 0.2, 0.3, 0.5, and 1.0 g) were immersed with 50 mL metal ions (50 ppm Cd^2+^, Pb^2+^) with constant pH 6 and 7 for Cd^2+^and Pb^2+^, respectively, and stirring for two hours. In the filtrate, lead and cadmium were measured by atomic absorption spectrophotometer.

#### Metal concentration

The effect of contact time between the adsorbent and the adsorbate on the effectiveness of the adsorption was investigated. 50 mL of various Cd^2+^ and Pb^2+^ concentrations (10, 20, 30, 50, 75, and 100 ppm) were mixed with 0.1 g of PANI/Msc and left to adjust at 25ºC for 2 h. In the filtrate, lead and cadmium were measured by atomic absorption spectrophotometer.

#### Contact time

Equilibrium time as important parameters show the need for time for metal removal in adsorption processes. 0.1 g of PANI/Msc was mixed with 50 mL (50 ppm Cd^2+^, Pb^2+^) with a suitable pH and solution temperature 25ºC for interval 30, 60, 120 and 180 min. At predetermined time intervals, in the filtrate, lead and cadmium were measured by atomic absorption spectrophotometer.

#### Solution temperature

The effect of adsorption temperature was investigated at different solution temperatures of 25, 35, 45 and 55ºC. 0.1 g of PANI/Msc was stirred with 50 ml metal ion (50 ppm Cd^2+^, Pb^2+^) with stabilization of all aforementioned parameters. The solution was filtered, and the metal ions concentration Cd^2+^and Pb^2+^ in the filtrate were measured by atomic absorption spectrophotometer.

## Results and discussion

### A comparative study of the PANI/Msc nanocomposites for removal of Pb^2+^ and Cd^2+^

Adsorption of Cd^2+^ and Pb^2+^ on the various prepared polymer muscovite nanocomposites (PANI/Msc1, PANI/Msc2, and PANI/Msc3) was investigated at the best adsorption conditions in order to compare the prepared nanocomposites. High levels of cadmium and lead were removed by PANI/Msc1 (75.6% and 72.6% for Cd^2+^ and Pb^2+^, respectively) more than the others, indicating that the optimal way for preparing polyaniline is to use a 1:1 ratio of polyaniline to nanomuscovite (Table 2). So, the nanocomposites (PANI/Msc1) will be used for further experiments.

**Table 2:** Removal percentage of lead and cadmium by PANI/Msc nanocomposite.

Polyaniline: Nanomuscovite ratio	% Pb removal	% Cd removal
1:1 [PANI/Msc1]	75.6%	72.6%
2:1 [PANI/Msc2]	67.3%	65.4%
1:2 [PANI/Msc3]	66%	64.7%

### Nanocomposite [PANI/Msc] characterization

#### XRD

**Fig. 1:**
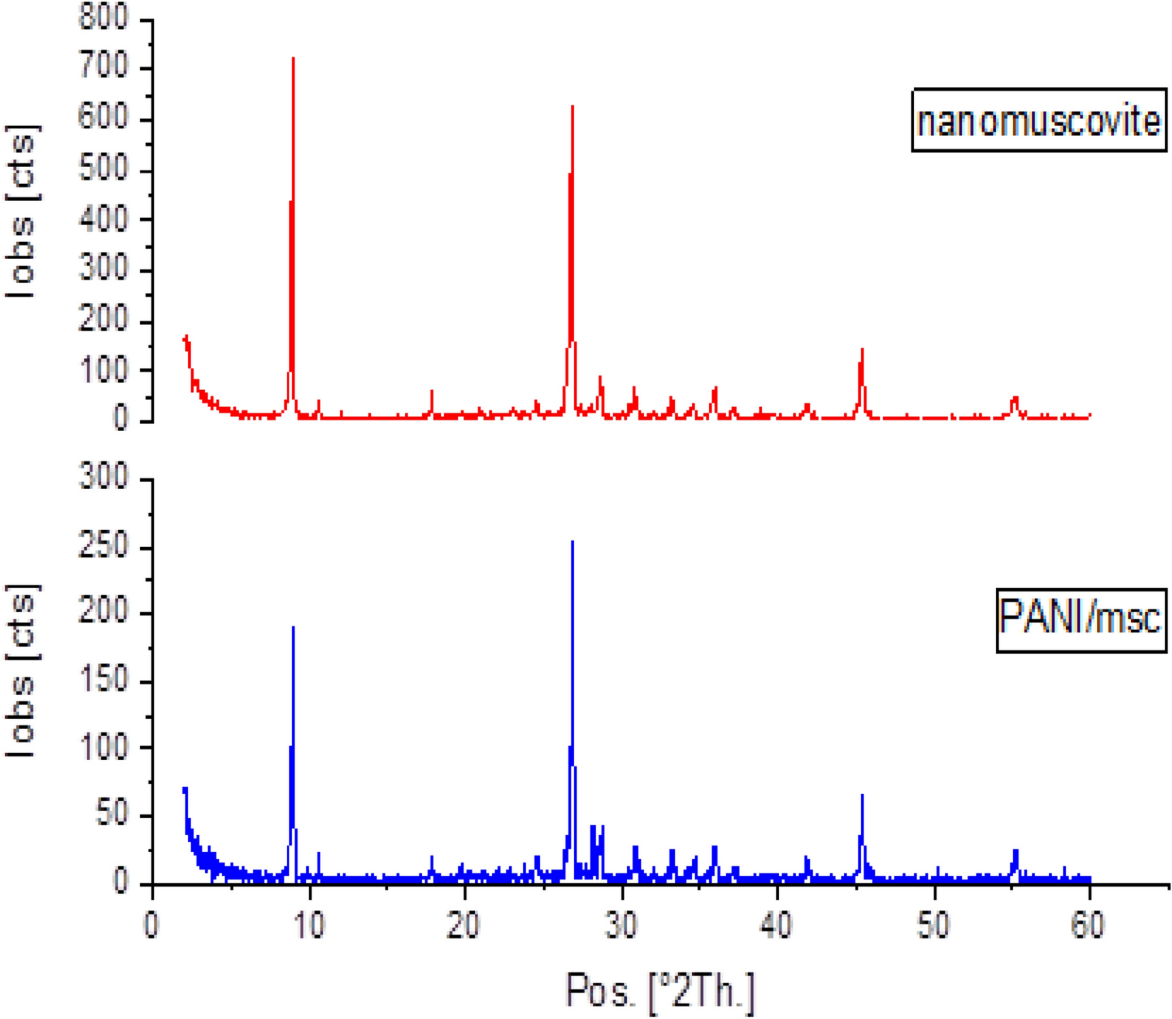
XRD of nanomuscovite and PANI/Msc1 nanocomposite.

The insertion of PANI chains into the nanomuscovite interlayer space was examined using XRD analysis of the nanocomposite [PANI/Msc1] using the 2Ө range of 0° to 60°. Figure 1 shows the XRD patterns of nanomuscovite and the PANI/Msc1 nanocomposite. This pattern shows a very small peak, approximately at 2θ = 28.8, for amorphous polyaniline due to its lower weight percent in the sample, and this illustrates the formation of the exfoliated state of PANI/Msc nanocomposite (Gholami et al., 2017). In this state, polyaniline chains increase the d-spacing of nanocomposite so that there is not a considerable interaction between silicate layers. Crystalline size was calculated using the Scherer equation and found in the range 15.6–45 nm.

#### SEM

**Fig. 2:**
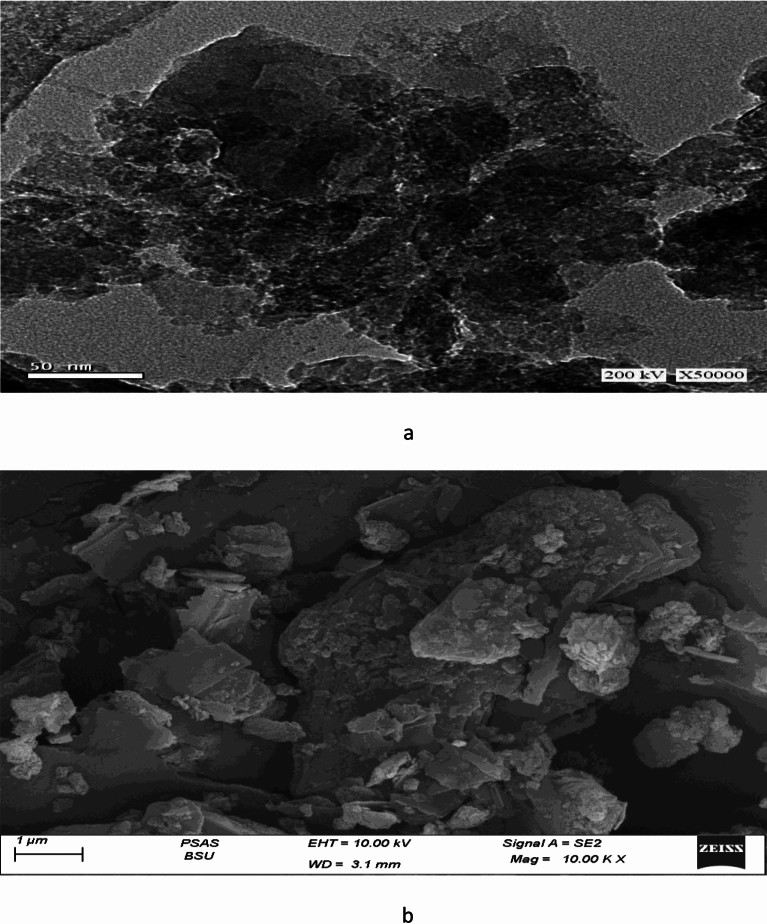
Images SEM of nanomuscovite (**a**), PANI/Msc1 nanocomposite (**b**).

SEM technique was used to morphological analyses and characterization of the size and shape of the resulted PANI/Msc1 nanocomposite. The granular texture and plate-like morphology of the Msc1 nanomuscovite particles were visible in the SEM images of the Msc1 nanocomposite (Fig. 2a). It is evident from Fig. 2b that the polymerization mostly took place between the Msc1 nanocomposite layers. Also, it confirmed that after modification of Msc1 nanomuscovite by PANI, the flaky Msc1 nanomuscovite structure coated by PANI and many individual platelets are seen in SEM images.This outcome demonstrates that PANI easily inserted itself between the Msc1 nanocomposite layers, expanded, and forced the layers apart, forming intercalated layered silicate PANI particles.

#### FTIR

**Fig. 3:**
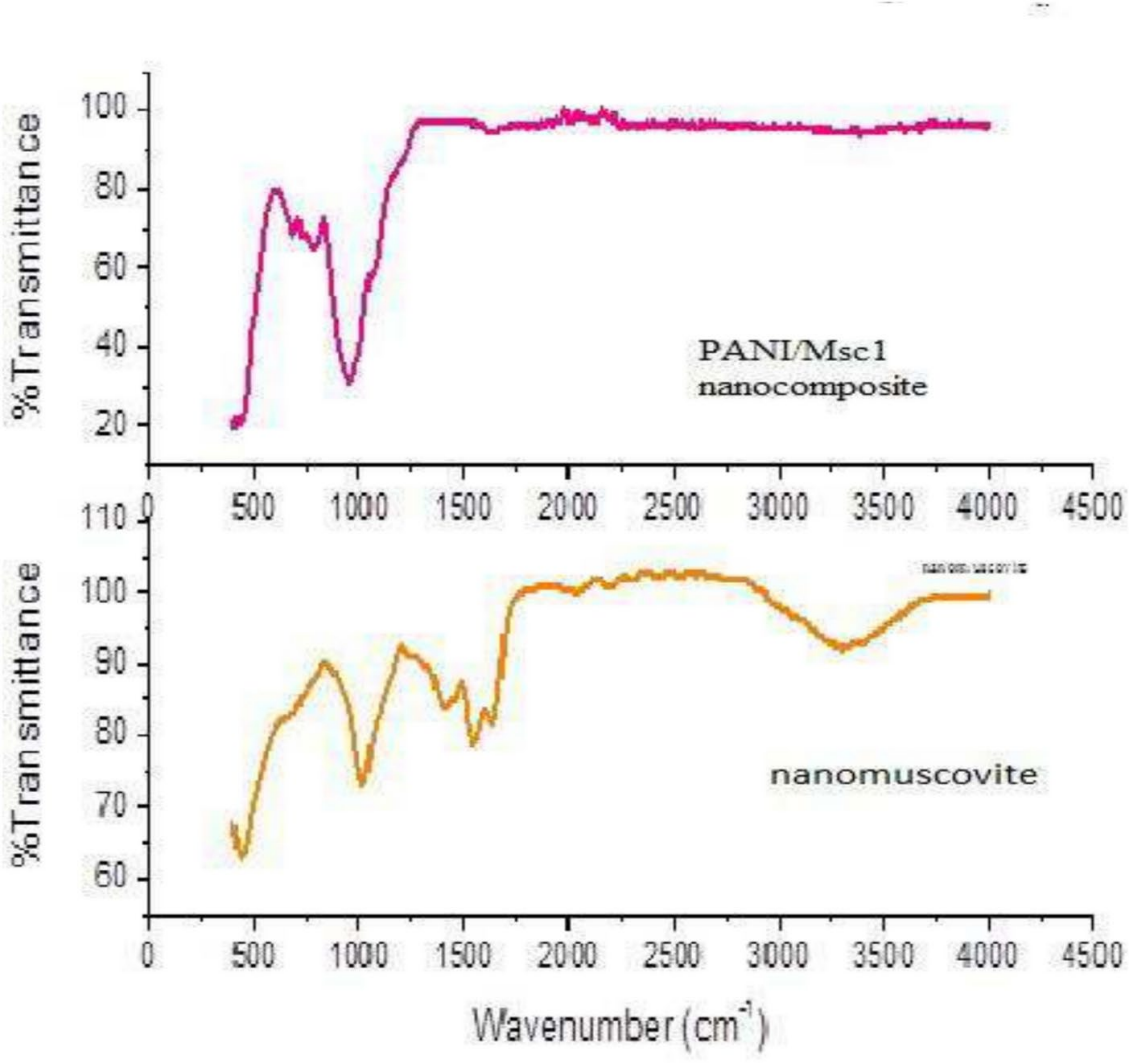
FTIR of nanomuscovite and PANI/Msc1 nanocomposite.

An FTIR spectrum of PANI/Msc1 nanocomposite and nanomuscovite is shown in Fig. 3. The nanomuscovite and PANI/Msc1 nanocomposite spectra show the peak at ~ 1016 cm^−1^, representing Si–O bond stretching. Bands at 2922 and 2855 cm^−1^ represented CH_2_ symmetric and asymmetric stretching vibration modes of alkyl chains.

The 440 cm^−1^ peak results from the Mg-O bending bonds. The Al-O bending bond bands may be detected at 918 cm^−1^. The hydration -OH vibration and HOH groups between the octahedral and tetrahedral sheets are attributed to the peaks at 1635 and 3298 cm^−1^^44^.

The 793 cm^−1^band is associated with the bending vibrations of OH^39^. The stretching vibrations of the CH_3_COO^-^ group, which occur symmetrically and asymmetrically, are responsible for the bands at 1542 cm^−1^ and 1403 cm^−1^, respectively. The band at 1395–1440 cm^−1^marks the emergence of interaction between the deformation vibration mode of the O–H group and the C-O vibration^45^. In the spectrum of the [PANI/Msc1] nanocomposite, the secondary amine is represented by a peak at 3,260 cm^−1^, and the benzenoid and quinoid rings of polyaniline are represented by peaks at 1,512 cm^−1^ and 1,592 cm^−1^, respectively.

#### Surface area

Nanomuscovite shows a surface area of 195.15 m^2^/g, while that for PANI/Msc1 nanocomposite was 208.626 m^2^/g (Table 3). These findings show that the PANI/Msc1 nanocomposite has a larger surface area than nanomuscovite. From Table 3, the pore size of nanomuscovite was 13.82 nm, and that for PANI/Msc nanocomposite was 13.74 nm. Due to the reduction in particle size, the pore size of nanocomposite was less than that of [PANI/Msc1] nanocomposite.

**Table 3:** Surface area and pore size of nanomuscovite and PANI/Msc1 nanocomposite.

Adsorbent	Surface area (m^2^/g)	Pore size (nm)	Pore volume (cm^3^/g)
Nanomuscovite	195.15	13.82	0.21
Polyaniline muscovite nanocomposite	208.6	13.74	0.25

### Optimum conditions for Cd^2+^ and Pb^2+^ ions adsorption on polyaniline muscovite nanocomposite [PANI/Msc1]

#### pH Effect

**Fig. 4:**
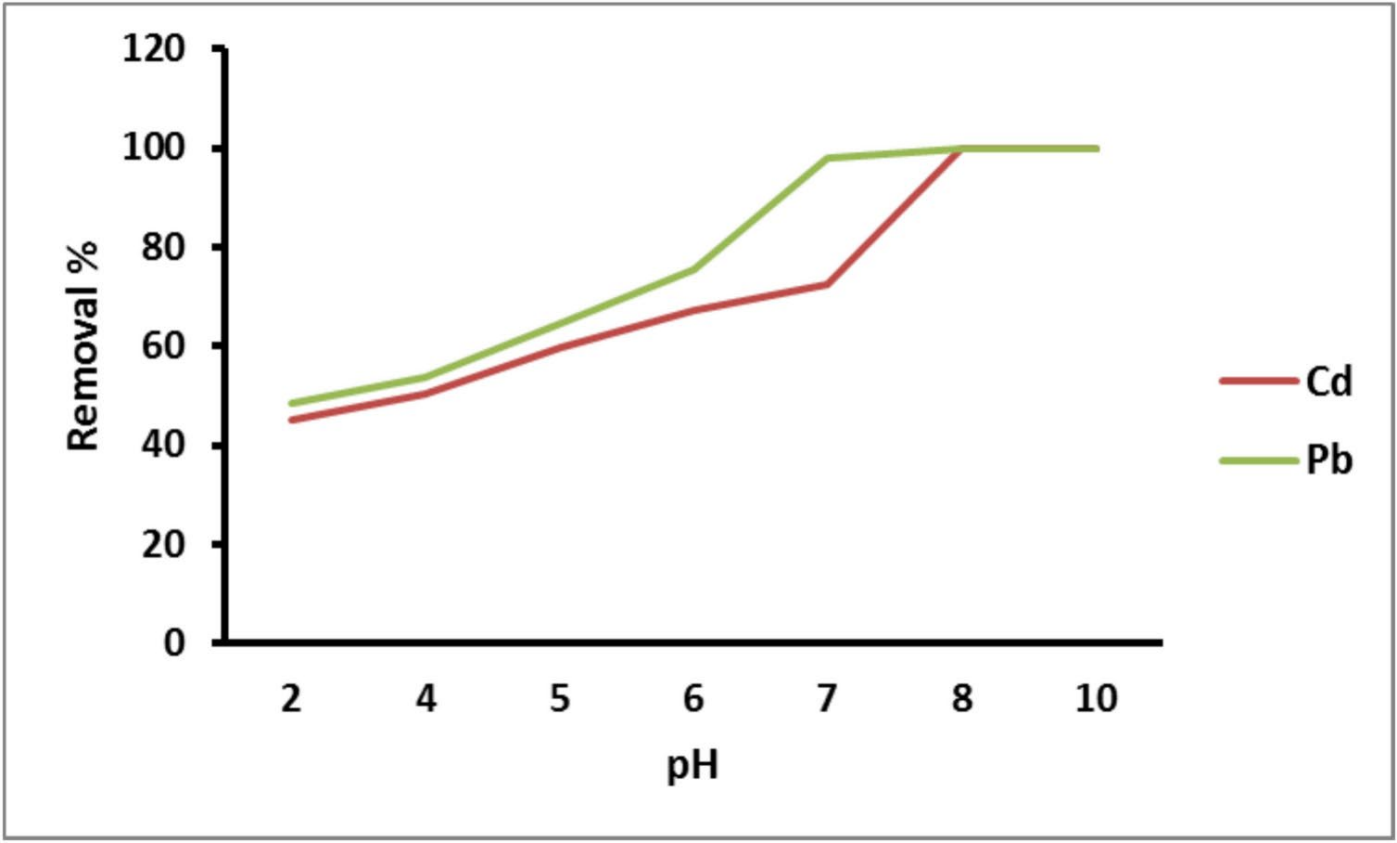
Removal of Pb^2+^ and Cd^2+^ on PANI/Msc1 nanocomposite according to pH at constant metal concentration 75 ppm, adsorbent dose 0.1 g, 25 oC solution temperature, and 60 min contact time.

Solution pH has a significant impact on the adsorbent’s surface functional groups and metal ion ionization degree as well as its water solubility. The effect of solution pH on the adsorption processes of Cd^2+^ and Pb^2+^ by the prepared PANI/Msc1 nanocomposite was studied with pH range (2, 4, 5, 6, 7, 8, and 10) is shown in Fig. 4. High metal removal of Pb^2+^ and Cd^2+^ was found at pH 7.0 (72.6%) and pH 6.0 (75.6%) for Cd^2+^ and Pb^2+^, respectively. Increased Pb^2+^ and Cd^2+^adsorption occurs at pH levels greater than 4 up to 7 due to more negatively charged the adsorbent and electrostatic repulsion reduction^46^. However, at pH levels over 7, Pb^2+^ and Cd^2+^adsorption rises to 100%; this may be because Pb and Cd hydroxide are poorly soluble in the solution and form an imperceptible precipitate^47^.

Piri, et al.^32^studied the removal of Pb(II) on polyaniline-modified clay nanocomposite and discovered that Pb was most effectively removed at a pH of 5.0. Gapusan and Balela^48^ reported the maximum removal of Pb ions from polluted water using polyaniline-kapok fibre nanocomposite at pH 4–6, which agreed with our results.

Mahmoud and Fekry^49^ studied the removal of divalent cadmium and lead on nanopolyaniline (NPANI) and cross-linked nanopolyaniline (CrossNPANI)/silica nanocomposites and found that the maximum capacity was at pH 6., which agreed with our results. Ahmad, et al.^50^ found that the highest amount of Cd (II) adsorbed from aqueous solutions on chitosan-grafted polyaniline-OMMT nanocomposite was at pH value 5.

#### Effect of adsorbent dose

**Fig. 5:**
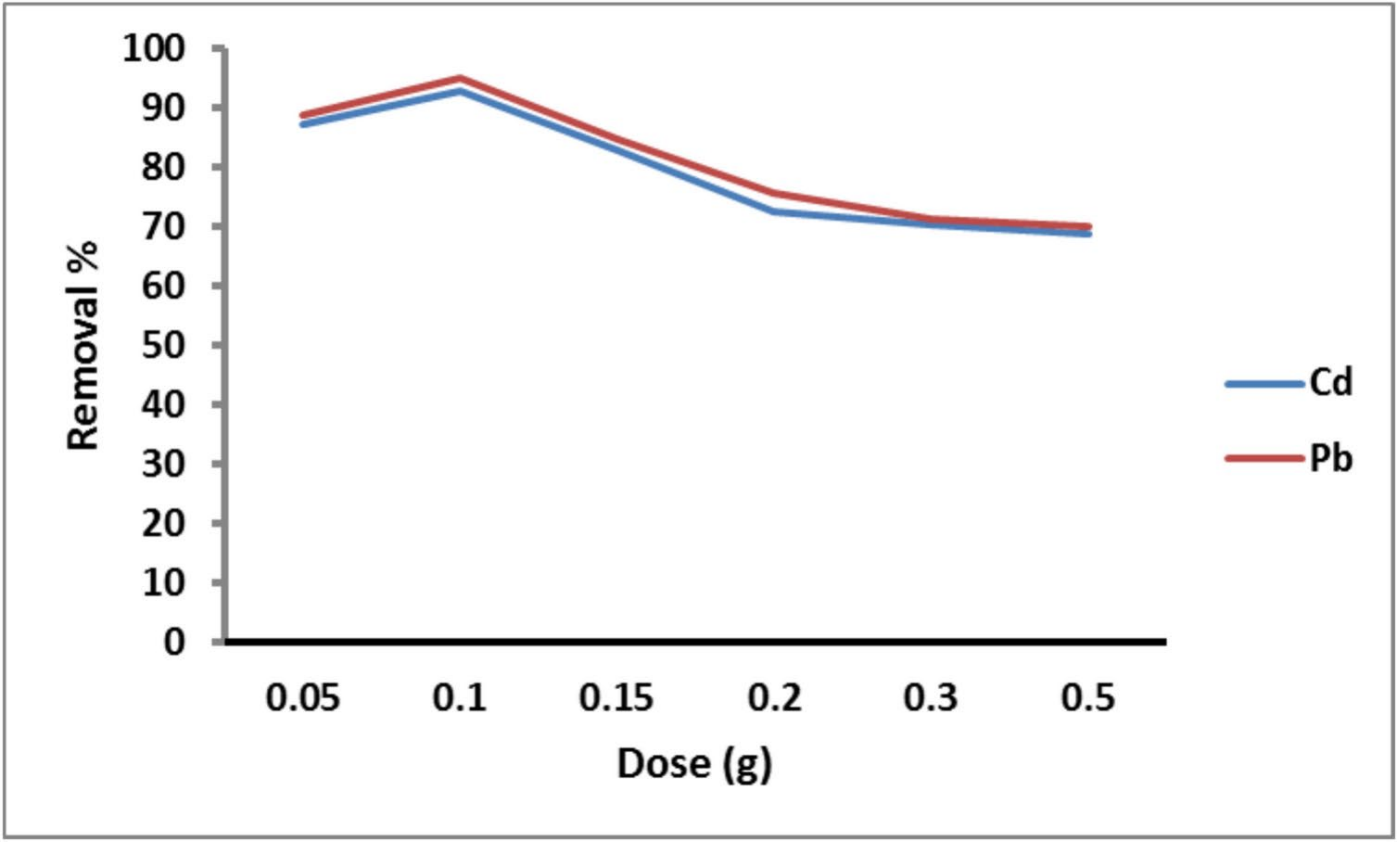
Removal of Cd^2+^ and Pb^2+^ on PANI/Msc1 nanocomposite according to dose at constant pH 6 and 7 for Pb^2+^ and Cd^2+^, metal concentration 75 ppm, 25 oC solution temperature, and 60 min contact time.

The removal of Cd^2+^ and Pb^2+^ ions versus the adsorbent dosage of PANI/Msc1 nanocomposite (0.05–0.5 g) is demonstrated in Fig. 5. The results show that the metal’s removal increases with increasing dose of adsorbent from 0.05 g to 0.1 g. The increasing metal adsorption on the adsorbent was related to increasing the active sites in the adsorbent because of the adsorbed equilibrium status between the solid and solution phase^51^. According to Hua^52^, the high adsorbent dose and the split in the concentration gradient between the adsorbent dose in the solution and on the adsorbent surface may result in shielding of binding sites due to screening impact on the impermeable outer layer. Rafiei, et al.^53^ reported that the maximum Pb removal efficiency (99.6%) on poly(acrylic acid)/bentonite nanocomposite was at 7.5 g L^−1^. Yadav, et al.^54^ studied the adsorption of Pb ions on clay-CNT nanocomposite in aqueous media achieved with an adsorbent dose (1.4 g L^−1^).

#### Effect of initial metals concentration

**Fig. 6:**
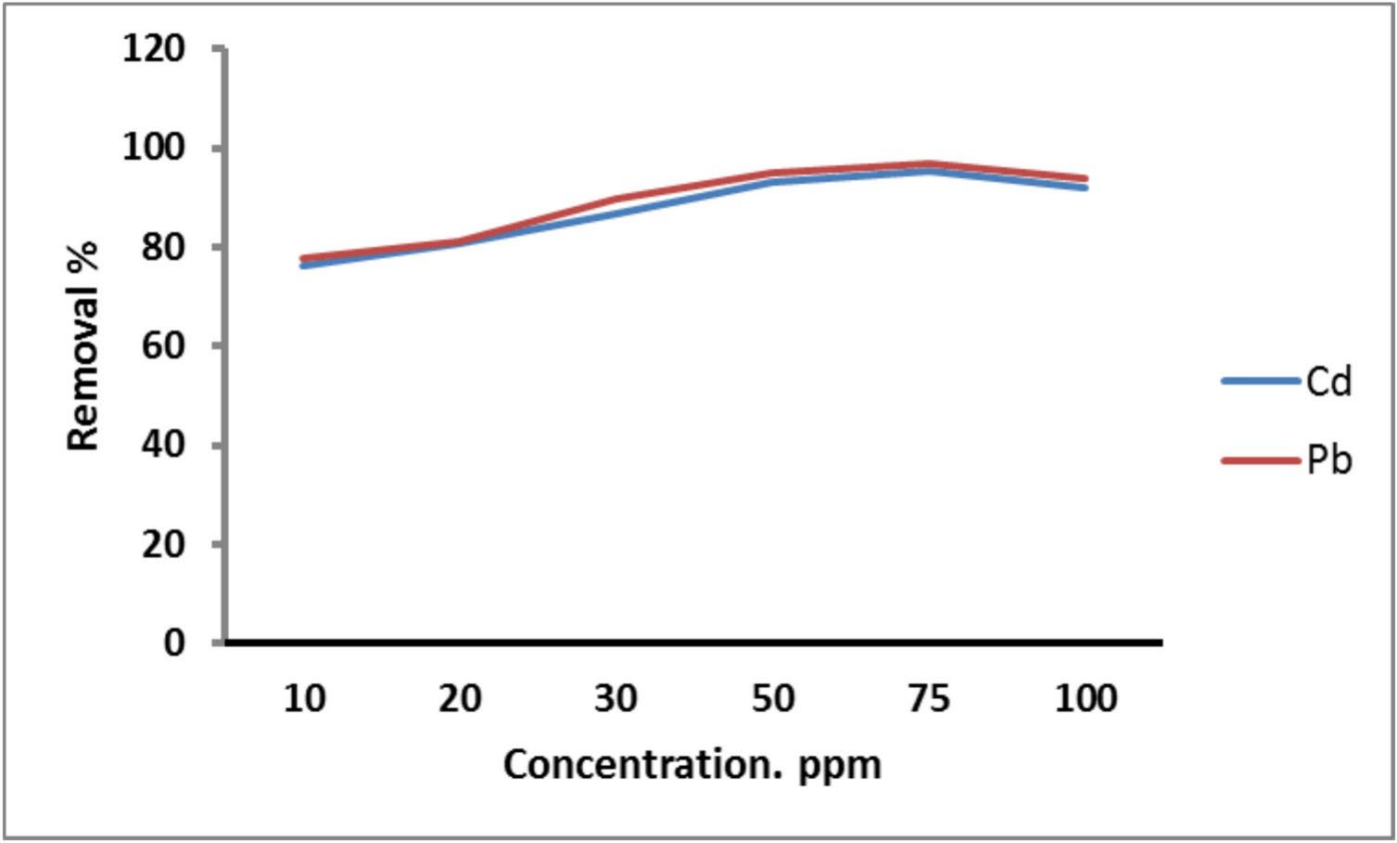
Removal of Cd^2+^ and Pb^2+^ on PANI/Msc1 nanocomposite according to initial metal concentration at constant pH 6 and 7 for Pb^2+^ and Cd^2+^, adsorbent dose 0.1 g, 25 oC solution temperature, and 60 min contact time.

Figure. 6 represents the relationship between the adsorption efficiency of Cd^2+^ and Pb^2+^ on PANI/Msc1 and the metal initial concentration. The results reveal that the removal percentage of Cd^2+^ and Pb^2+^ increased gradually with the metal concentration and reached the maximum adsorption (95.5% and 97% for Cd^2+^ and Pb^2+^, respectively) at 75 ppm. So, Cd^2+^ and Pb^2+^concentration (75 ppm) was used for further experiments. The amount of metal adsorbed increased with increasing the metal concentration as a result of the rising driving force of metal ions onto the adsorbent active sites^12^.

Lead adsorption on clay-CNT nanocomposite in aqueous media was reported by Yadav et al. (2019), who ended that maximum Pb adsorption was at an initial lead concentration of 15 mg L^−1^**.** Piri, et al.^32^ studied the removal of Pb(II) ions on polyaniline-modified clay nanocomposite and found that the optimum metal adsorption was at 200 mg L^−1^ initial Pb(II) concentration.

#### Effect of contact time

**Fig. 7:**
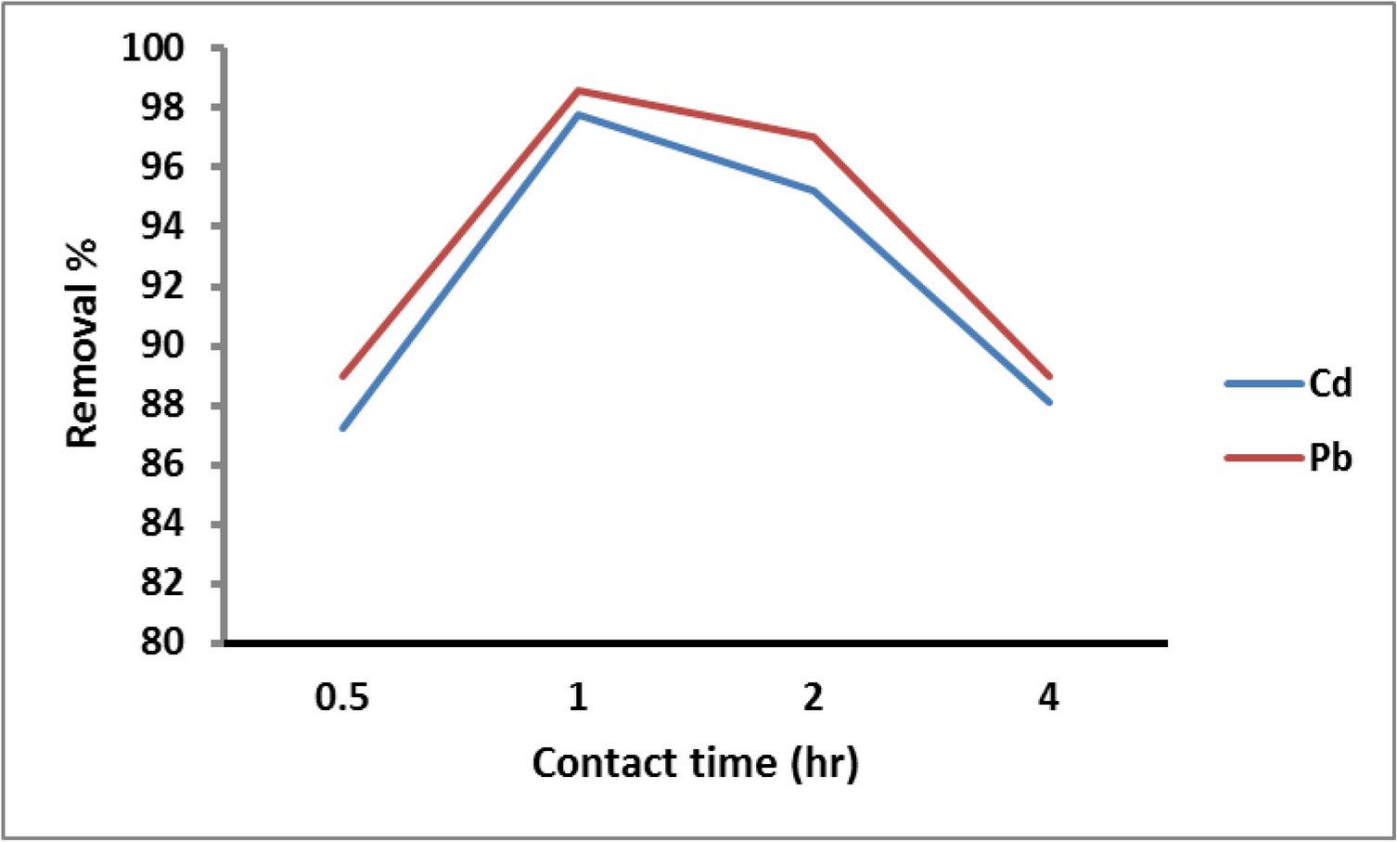
Removal of Cd^2+^ and Pb^2+^ on PANI/Msc1 nanocomposite according to contact time at constant metal concentration 75 ppm, pH 6 and 7 for Pb^2+^ and Cd^2+^, adsorbent dose 0.1 g, and 25 oC solution temperature.

The effect of the contact time on the removal of Cd^2+^ and Pb^2+^ by PANI/Msc nanocomposite adsorbent is illustrated in Fig. 7. The removal efficiency of Cd^2+^ and Pb^2+^ ions by the PANI/muscovite nanocomposite increases as the contact time increase and reach the maximum metal adsorption at 60 min (87.2% and 89% of Cd^2+^ and Pb^2+^ ions, respectively). Rapid Cd^2+^ and Pb^2+^adsorption on PANI/Msc1 nanocomposite may be due to the abundance of active sites on the adsorbent. The removal process is slowed after 60 min due to the progressive decline in the number of active sites, which causes the remaining unoccupied sites to be difficult to access due to repulsive forces between the metal ions on the solid surface and the bulk phase^55^.

Karimi, et al.^56^ reported that the optimum time for the maximum adsorption of Pb (II) on polythiophene/Al_2_O_3_ and its modified poly(vinyl alcohol) was 40 min. Pb(II) ion adsorption was investigated by Msaadi, et al.^57^ on a polymer/clay nanocomposite, and it was discovered that equilibrium was reached in 15 min. Rafiei, et al.^53^ reported that Pb ion removal on poly(acrylic acid)/bentonite nanocomposite was maximum at 30 min. Yadav, et al.^54^ studied Pb ions adsorption on clay-CNT nanocomposite was achieved within 120 min.

#### Effect of temperature

**Fig. 8:**
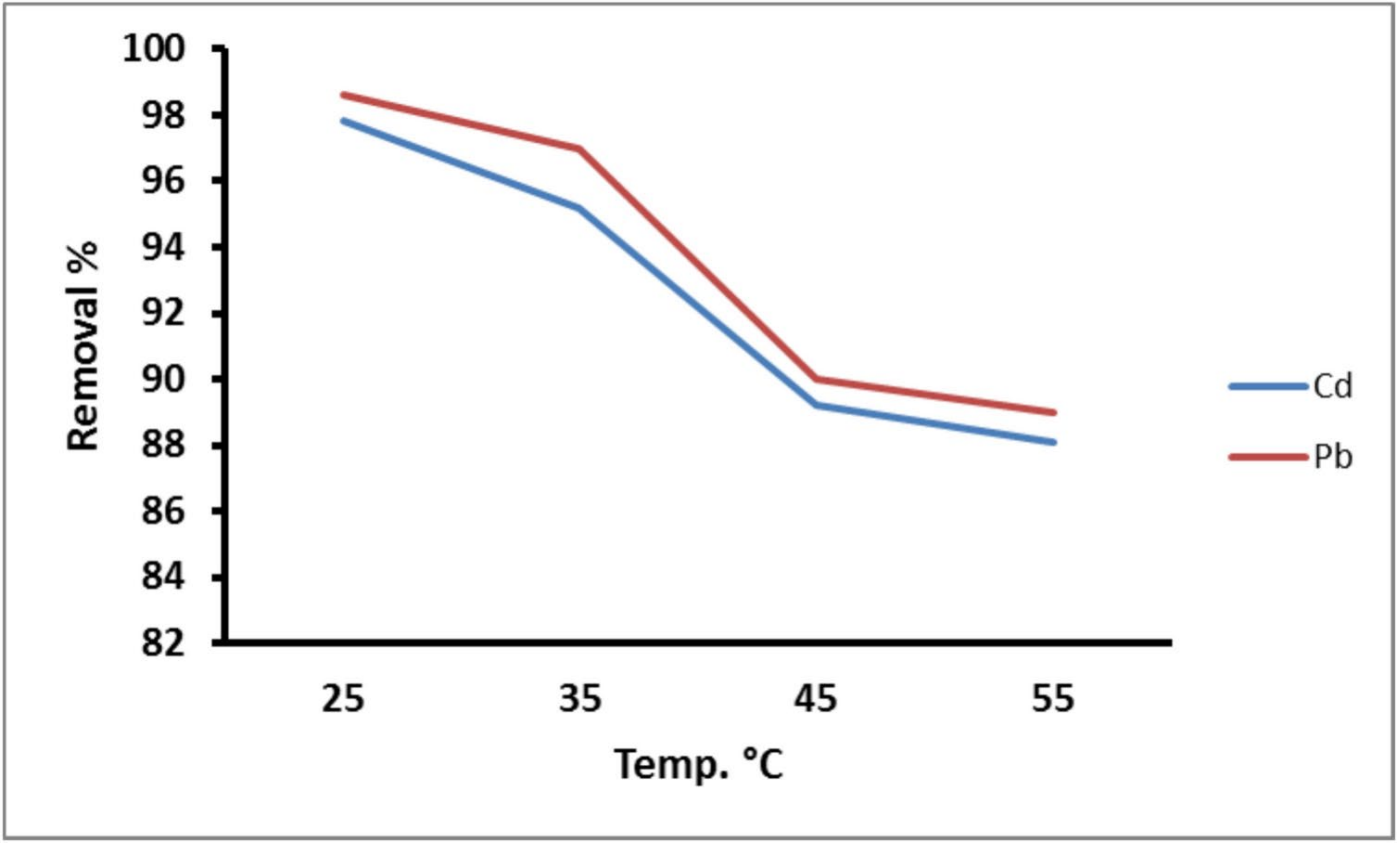
Removal of Cd^2+^ and Pb^2+^ on PANI/Msc1 nanocomposite according to temperature at constant metal concentration 75 ppm, pH 6 and 7 for Pb^2+^ and Cd^2+^, adsorbent dose 0.1 g, and 60 min contact time.

The results (Fig. 8) of solution temperature on the adsorption of the metals showed that the percentage adsorption efficiency of Cd^2+^ and Pb^2+^was high at 25 °C (97.8% and 98.8% for Cd and Pb, respectively); after that, it diminishes as a result of the force of the van der Waals bonds breaking down, resulting in a reduction of active sites^58^.

### Adsorption isotherms

The adsorption isotherms parameters (Langmuir, Freundlich, Temkin, and Dubinin-Raduskevich (D-R) isotherms) of Cd^2+^ and Pb^2+^ on PANI/Msc1 nanocomposite are presented in Table 4.

**Table 4:** Adsorption isotherms parameters for Cd^2+^ and Pb^2+^ adsorption on PANI/Msc1 nanocomposite.

Models	Parameters	Pb	Cd
Langmuir model	Q_0_ (mg/g)	36.1	61
	b_L_ (L/mg)	0.05	0.39
	R^2^	0.98	0.999
Freundlich model	1/n	0.264	0.363
	K_f_ (mg^-1^/n L^1/n^ g^-1^)	2.21	2.09
	R^2^	0.78	0.809
Temkin model	B (j/mol)	14.1	12.44
	bT (j/mol)	177.5	201.2
	Kt (L/g)	1.12	1.84
Dubin-Raduskevich model	qD (mg/g)	20.9	7.4
	E (kj/mol)	0.6	0.35
	R^2^	0.82	0.91

For adsorption isotherm and kinetic models to evaluate the goodness of the estimates, we used the following established criteria:- R^2^ > 0.9: Indicates a good fit of the model to the experimental data.

#### Freundlich isotherm

To determine how heterogeneous surface energy is consumed during adsorption, the Freundlich isotherm is applied. The practical Freundlich formula is:

3 $$\text{Log }{\text{q}}_{\text{e}}={\text{logK}}_{\text{f}}+\frac{1}{\text{n}}{\text{logC}}_{\text{e}}$$

The adsorption intensity and capacity are represented by the constants n and K_f_, respectively. From the equation, linear relationship’s slope and intercept between log q_e_ and log C_e_ can be utilized to calculate K_f_ and n. The information is displayed in Table 4. As a result, 1/n values less than 1 imply normal adsorption with a significant contact between the adsorbent and the metal, whereas cooperative adsorption is indicated by 1/n values more than 1.

The results show that the values for Cd^2+^ K_f_ and 1/n were 0.363 and 2.1, while those for Pb^2+^ were 0.264 and 2.3, respectively. The results reveal that 1/n value was less than 1, which indicates that the adsorption of Cd^2+^ and Pb^2+^ onto PANI/Msc1 nanocomposite was favorable. The R^2^ values were 0.805 for Cd^2+^ and 0.773 for Pb^2+^ lower than those with Langmuir, which indicated that this isotherm did not fit this adsorption.

#### Langmuir isotherm

The saturated monolayer adsorption on the uniform surface was characterized using the Langmuir isotherm model at constant energy. The equation may quantify the Langmuir equation.:

4 $${{{\text{c}}_{{\text{e}}} } \mathord{\left/ {\vphantom {{{\text{c}}_{{\text{e}}} } {{\text{q}}_{{\text{e}}} }}} \right. \kern-0pt} {{\text{q}}_{{\text{e}}} }} = {1 \mathord{\left/ {\vphantom {1 {{\text{Q}}_{{\text{o}}} }}} \right. \kern-0pt} {{\text{Q}}_{{\text{o}}} }}{\text{b}}{\kern 1pt} { + }{{{\text{c}}_{{\text{e}}} } \mathord{\left/ {\vphantom {{{\text{c}}_{{\text{e}}} } {{\text{Q}}_{{\text{o}}} }}} \right. \kern-0pt} {{\text{Q}}_{{\text{o}}} }}$$

*C*_e_, the metals’ equilibrium concentration (ppm), Q_o_ for capability of monolayer adsorption (mg/g), *q*_e_ for the adsorbent’s metal equilibrium capacity (mg/g), and b for the coefficient of binding energy in Langmuir’s model (L/mg). From the equation, the slope and intercept of C_e_/q_e_ vs. C_e_ may be used for computations b and Q_o_. The information is displayed in Table 4.

The correlation coefficients (R^2^) of Cd^2+^ and Pb^2+^ on PANI/Msc1 nanocomposite were determined from the data in Table 4 to be 0.999 and 0.98, respectively, which suggested that the adsorption mechanism is of a monolayer.

Furthermore, it was discovered that Cd^2+^ and Pb^2+^ each had a maximum monolayer adsorption capacity (Qo) of 61 and 36.1 mg g^−1^, respectively. The sorption of Cd^2+^ and Pb^2+^ ions on PANI/Msc1 nanocomposite is favorable according to the Langmuir isotherm, which is also supported by the results b_L_ < 1 for the concentration examined. Since the Langmuir equation presumes surface homogeneity, the finding that the Langmuir model best matches the experimental results may be related to the uniform distribution of active sites on the adsorbent surfaces.

The maximum adsorption capacity of PANI/Msc1 nanocomposite for Cd^2+^ and Pb^2+^ ions was compared with values of different adsorbents reported in the literature, as presented in Table 5

**Table 5:** Comparison of the maximum adsorption capacity of PANI/Msc1 nanocomposite for Cd^2+^ and Pb^2+^ ions with other adsorbents reported in the literature.

Adsorbent	Qm (Pb^2+^) (mg/g)	Qm (Cd^2+^) (mg/g)	Kinetics/Isotherm	Ref
Activated muscovite	12.95	13.12	Pseudo second-order/ Langmuir isotherm	^35^
Nanomuscovite	25.5	26.6	Pseudo second-order/ Langmuir isotherm	^37^
Magnetic Biochar	26.3	23.6	Pseudo second-order/ Langmuir isotherm	^10^
Wetland plant reed/NaBH4, FeSO_4_	38.31	39.35	Pseudo second-order/ Langmuir isotherm	^59^
Magnetic biochar composite	25.29	14.96	Pseudo second-order/ Langmuir isotherm	^60^
activated coal bottom ash	5.83	2.72	Pseudo second-order/ Langmuir isotherm	^61^
natural kaolinite clay	2.35	1.35	Pseudo second-order/ Langmuir isotherm	^62^
MWCNT-coated polyaniline	22.2	–––	Pseudo second-order/ Langmuir isotherm	^63^
Polyaniline/clay	70.4	––-	Pseudo second-order/ Langmuir isotherm	^32^
natural clay modified by polyvinylpyrrolidone	0.67	0.78	Pseudo second-order/ Langmuir isotherm	^64^
PANI/Msc nanocomposite	36.1	61	Pseudo second-order/ Langmuir isotherm	This study

#### Temkin isotherm

Temkin isotherm model predicts that the binding energies of adsorbents on a surface will be uniformly distributed throughout the population.

The linear form of the Temkin equation is given by the following:

5 $${\text{q}}_{{\text{e}}} = {\text{B1n}}{\kern 1pt} {\text{K + B1n}}{\kern 1pt} {\text{c}}_{{\text{e}}}$$

$${\text{B}}{\kern 1pt} { = }{{{\text{RT}}} \mathord{\left/ {\vphantom {{{\text{RT}}} {\text{b}}}} \right. \kern-0pt} {\text{b}}}$$

The sorption heat is denoted by the Temkin constant b. The constants B, associated with the heat of sorption (J/mol), and K, associated with Temkin equilibrium binding constant, can be calculated from a plot of q_e_ vs. ln C_e_. From the Temkin plot, the Cd^2+^ and Pb^2+^ adsorption constant values were K_T_ 1.84 and 1.12 L g^−1^, R^2^ = 0.94 and 0.94, and B = 12.44 and 14.1 J mol^−1^, respectively.

#### The isothermal Dubinin-Radushkevich model

The Dubinin-Radushkevich isotherm is commonly used to explain the adsorption mechanism with Gaussian energy distribution over a heterogeneous surface. It is employed to distinguish between chemical and physical metal adsorption. The linearized D-R equation:

6 $${\text{Ln q}}_{{\text{e}}} = {\text{ln q}}_{{\text{m}}} { }{-}{\text{ BE}}^{2}$$

It may determine the values from the slope and the qm values from the intercept by plotting ln q_e_ vs. E^2^. Regarding both chemical and physical adsorption, information is provided by the mean adsorption energy (E).

As seen in Table 4, the calculated mean free energy (E) values for Pb^2+^ and Cd^2+^ are 0.5 kJ/mol and 0.32 kJ/mol, respectively. The type of Cd^2+^ and Pb^2+^ adsorption onto PANI/Msc1 nanocomposite was characterized as physical adsorption because it was found that (E) for Cd^2+^ and Pb^2+^ is lower than the range of adsorption reaction 8–16 kJ mol^−1^.

Compared to the other Isotherm models, the Cd^2+^ and Pb^2+^ adsorption on PANI/Msc1 nanocomposite suited Langmuir isotherm best (Table 4).

### Adsorption kinetic

The mechanism of the adsorption process has been determined using kinetic models^65^, which gives significant information to increase adsorption efficiency and the viability of process scaling up.

The sorption kinetics of Cd^2+^ and Pb^2+^ on polyaniline muscovite [PANI/Msc1] nanocomposite adsorbent were investigated using the Pseudo-first order, Pseudo-second order, Elovich, and intra-particle diffusion models.

All these adsorption steps, including adsorption, internal particle diffusion, and external film diffusion, are incorporated into these models. Table 6 represents the Cd^2+^ and Pb^2+^ adsorption kinetic data on the polyaniline muscovite [PANI/Msc1] nanocomposite.

**Table 6:** Adsorption kinetic parameters of Cd^2+^ and Pb^2+^ on PANI/Msc1 nanocomposite.

Models	Parameters	Pb	Cd
Pseudo first-order	K^1^ (min)^−1^	0.01	0.02
	q_e_ (mg/g)	4.17	2.84
	R^2^	0.73	0.61
Pseudo second-order	K_2_ (g/mg min)	6.2*10^–3^	6.7*10^–3^
	q_e_ (mg/g) (calculated)	33.1	32.8
	q_e_ (mg/g) (experimental)	36.4	35.7
Elovich model	α (mg/min)	6.8	7.1
	β (g/mg)	0.46	0.46
	R^2^	0.64	0.62
Intra particle diffusion model	K_i_	0.5	0.49
	C (mg g^-1^)	31.5	31.1
	R^2^	0.723	0.708

#### Pseudo–first order model

The pseudo-first-order kinetic is depicted in the following model:

7 $${\text{Log }}\left( {{\text{qe}}_{{\text{e}}} { }{-}{\text{ q}}_{{\text{t}}} } \right) = {\text{log q}}_{{\text{e}}} { }{-}{ }\left( {{\text{K}}_{1} /2.303} \right){\text{ t}}$$

The pseudo-first order (*K*_1_) is the rate constant, and the adsorption capacities at equilibrium and t (in minutes) are q_e_ and q_t_ (in mg/g), respectively.

By plotting log (q_e_ − q_t_) vs. (t), the kinetic constants for the pseudo-first order adsorption of Cd^2+^ and Pb^2+^ on PANI/Msc1 nanocomposite were obtained from the slope and the intercept (Table 6). The results revealed the correlation coefficient (R^2^) were 0.61 and 0.73 for Cd^2+^ and Pb^2+^ onto PANI/Msc1 nanocomposite, respectively, while k_1_ values were 0.02 and 0.01 min^−1^.

In this model (The model of pseudo first order) R^2^ value for Cd^2+^ and Pb^2+^ is lower than those in other models, implying that the model is not applicable and that there is not a great deal of agreement between the theoretical and experimental q_e_ values. This suggests that the pseudo-first order model may not be sufficient to understand how Cd^2+^ and Pb^2+^ interact with PANI/Msc1 nanocomposite.

#### Pseudo–second order model

A depiction of the pseudo-second order kinetic model is as follows:

8 $${\text{t}}/{\text{q}}_{{\text{t}}} = 1/{\text{K}}^{2} {\text{q}}_{{\text{e}}}^{2} { } + { }1/{\text{q}}_{{\text{e}}} {\text{t}}$$

K^2^, the rate constant (g/mg/min).

By charting t/qt vs. time (t) using the equation, q_e_, and K^2^ can be calculated from the intercept and slope. The results show high correlation coefficients (R^2^) of 0.998 and 0.997 for Cd^2+^ and Pb^2+^ ions, respectively (Table 6). Also, the estimated adsorption capacity at equilibrium (q_e_) were 32.8 and 33.1 mg g^−1^ for Cd^2+^ and Pb^2+^, respectively. The values q_e_ cal were near q_e_ exp values, which indicates that the adsorption of Cd^2+^ and Pb^2+^ ions behaved in a pseudo-second-order approach.

#### Intra-particle diffusion model

An intra-particle diffusion model is frequently used to characterize solid–liquid adsorption’s solute transfer, and it was utilized to identify the adsorption mechanism.

9 $${\text{q}}_{t} = {\text{K}}_{i} {\text{t \raise.5ex\hbox{$\scriptstyle 1$}\kern-.1em/ \kern-.15em\lower.25ex\hbox{$\scriptstyle 2$} }} + {\text{ C}}$$

*K*i (mg g^−1^ min^−1/2^) is the intra-particle diffusion rate constant, while *C* (mgg^−1^) indicates the boundary layer effect and thickness.

**Fig. 9:**
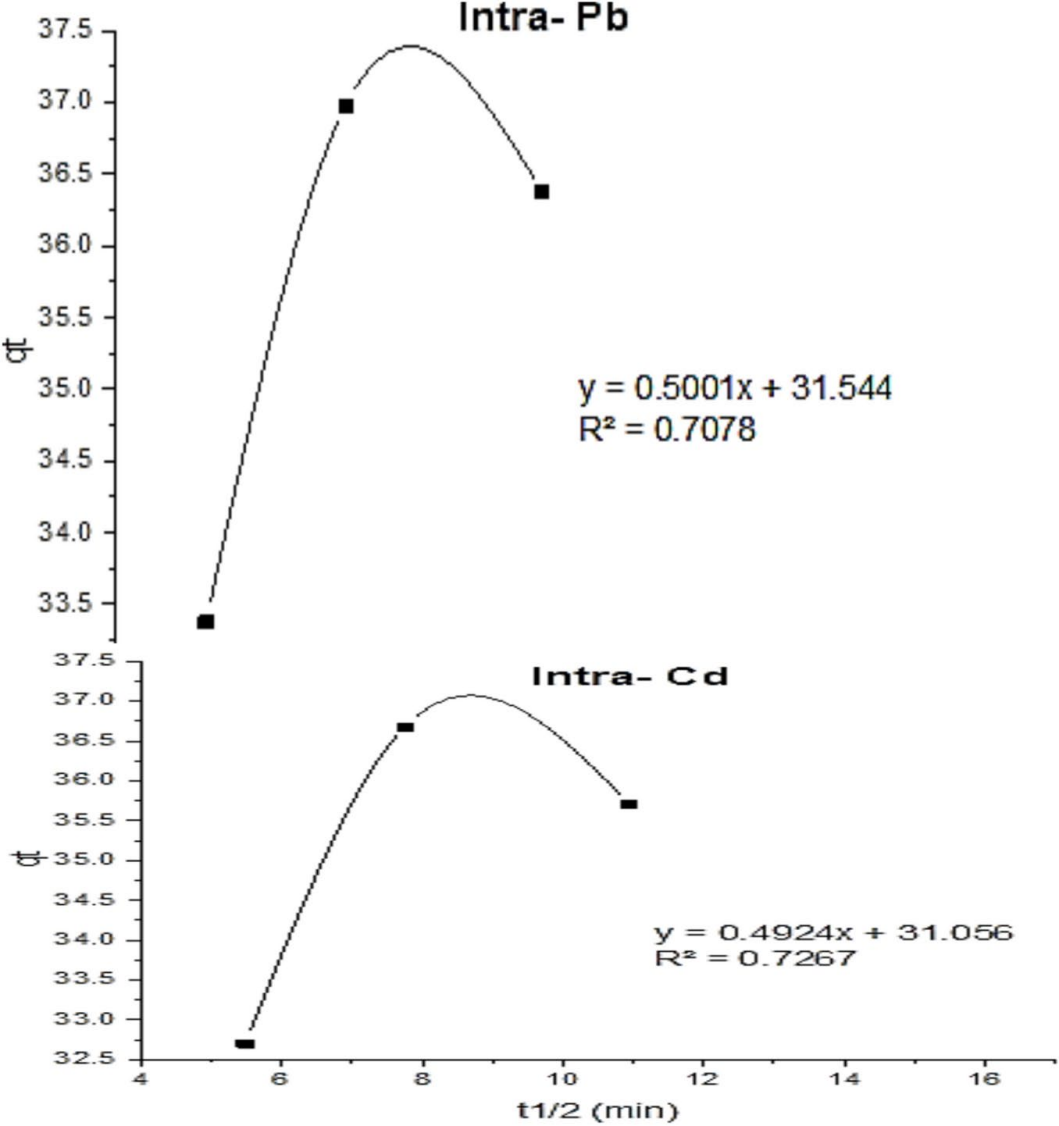
The intra-particle kinetics of Cd^2+^ and Pb^2+^ adsorption on Polyaniline Muscovite [PANI/Msc] nanocomposite.

From the equation, the slope and intercept of the linear plot of qt vs. t^1/2^ can be used to get the K_i_ and C (Fig. 9). Intraparticle diffusion constants were calculated and are presented in Table 5 for the sorption of Cd^2+^ and Pb^2+^. K_id_ values were 0.49 and 0.5 mg g^−1^ min^−1/2^ for Cd^2+^ and Pb^2+^, respectively, while R^2^ were 0.708 and 0.723, respectively. The C values (31.5 and 31.1 mg g^−1^).

Figure 9depicts multilinearity forms, and this may involve two or more additional phases. The first demonstrates that the greater sharpness of the line is due to the propagation of divalent ions from the solution to the adsorbent’s exterior surface or to the diffusion of solute ions through boundary strata. Intraparticle diffusion is the rate-limiting process during the progressive adsorption stage^66–68^.

#### Elovich model

Elovich equation characterizes the activated chemisorption:

10 $$q_{t} = 1/\beta \ln \left[ {\alpha \beta } \right] + 1/\beta \ln t$$

α, the initial adsorption, and *β,* the adsorption coefficient.

The constants α and β were obtained from the equation by plotting q_e_ vs. ln(t) and represented in Table 6. The α values were 7.1 and 6.8 mg min^−1^ for Cd^2+^ and Pb^2+^, respectively, while β values were 0.46 and 0.46 for Cd^2+^ and Pb^2+^, respectively. A plot of q_t_ versus ln t shows that the adsorption of Cd^2+^ and Pb^2+^ onto PANI/Msc nanocomposite did not obey the Elovich equation due to small R^2^ values.

According to the Elovich model, the rate-controlling step (RCS) is the chemical reaction between Cd^2+^ and Pb^2+^and the surface of the adsorbent and the removal procedure is carried out by multiple-layer adsorption^69^.

### Thermodynamics of adsorption

Several indicators for particle applications are entropy (ΔS°), free energy (ΔG°), and enthalpy (ΔH°). In this study, the thermodynamics of Cd^2+^ and Pb^2+^ adsorption on the prepared adsorbent were evaluated in light of various temperatures (298, 313, and 333 K).

For the purpose of calculating the thermodynamic parameters, the following equation was utilized^70^.

11 $$Ln K = \Delta S^{o} /R {-} \Delta H^{o} /RT$$

*R,* and *T* are constants related to gas (8.314 J/mol K) and temperature (K), respectively.

The equation below determines the free energy of specific adsorption ΔG^o^ (KJ mol^−1^).

12 $$\Delta G^{o} = \Delta H^{o} {-} T \Delta S^{o}$$

Table 7 lists the estimated thermodynamic parameters.

**Table 7:** Cd^2+^ and Pb^2+^ adsorption thermodynamic parameters.

Parameters		Cd^2+^	Pb^2+^
ΔH^o^, kJ/mol		−15.2	−16.4
ΔS^o^, kJ/mol K		−0.174	−0.183
ΔG^o^, kJ/mol	298 K	−7.4	−8.9
	308 K	−5.9	−7.04
	318 K	−4.5	−5.2
	328 K	−3	−0.3.4
R^2^		0.939	0.932

The results reveal that G° values for Cd^2+^ and Pb^2+^ were negative at all temperatures, which points to a physical adsorption mechanism for the adsorption of Cd^2+^ and Pb^2^ on PANI/Msc1 nanocomposite. In order to indicate a favourable and spontaneous absorption of Cd^2+^ and Pb^2+^, the free energies ΔG° of the PANI/Msc1 nanocomposite were calculated in negative signs. ΔH° parameter shows negative values, which indicate that adsorption is exothermic, and the low value of ΔH° indicates that this adsorption system might function primarily via physical adsorption (Draman et al. 2014). When Cd^2+^ and Pb^2+^ were adsorbed on the active sites of the adsorbent surface, the negative ΔS° indicated enhanced randomness at the solid-solution interface.

## Adsorption mechanism of Cd^2+^ and Pb^2+^ on PANI/Msc1 nanocomposite

The adsorption mechanism of Cd^2+^ and Pb^2+^on PANI/Msc1 nanocomposite involves several steps. Firstly, the nano Muscovite (Msc1) serves as a growth template, allowing the Polyaniline (PANI) to form a nanocomposite with enhanced surface area and reactivity^71^. Generally, the adsorption of heavy metal ions onto PANI/Msc1 nanocomposite occurs through electrostatic interactions and ion exchange mechanisms between the heavy metal ions and the adsorbent. The PANI component plays a crucial role in the adsorption process, as it provides amino and imino functional groups that can chelate with Cd^2+^ and Pb^2+^ ions. It is very clear where nitrogen atoms exist in amine compounds due to the presence of electron in SP3 orbital of nitrogen can makes coordinate bond with positive charge of analytes (Cd^2+^, Pb^2+^). This chelation process is facilitated by the electrostatic attraction between the positively charged Pb and Cd ions and the negatively charged PANI chains^32^.

## Conclusion

A novel form of polyaniline/muscovite nanocomposite (PANI/Msc1) was synthesized from nanomuscovite by polymerization of aniline on the surface of nanomuscovite. The adsorption of Cd^2+^ and Pb^2+^ onto the PANI/Msc1 nanocomposite was found to be more favourable at pH 6 and 7 for Pb^2+^ and Cd^2+^, respectively, with an adsorbent dose 0.1 g, metal concentration 75 ppm, solution temperature 25 °C, and contact time 60 min. Adsorption isotherm and kinetics show that the adsorption of Pb^2+^ and Cd^2+^ agrees with the Langmuir isotherm model and follows the pseudo-second order kinetic model. Furthermore, thermodynamic studies reveal that adsorption processes are both endothermic and spontaneous.

The prepared novel nanocomposite can be used successfully for the treatment of polluted water from heavy metal pollution. The synthesis process of the PANI/Msc1 nanocomposite reduces the overall cost of the adsorbent material, and can be scaled up for industrial production, making it a viable option for large-scale wastewater treatment applications.

The preparation of PANI/Msc1 nanocomposite offers significant advantages as it greatly enhances its surface and morphological properties, thereby improving its capacity to adsorb heavy metals. Also, the incorporation of nanomuscovite into the PANI matrix enhances the mechanical strength and improves thermal stability. The use of naturally abundant muscovite reduces the overall cost of the nanocomposite.

Our future research will focus on the application of PANI-Muscovite nanocomposite for the removal of other pollutants, such as dyes, pesticides, or pharmaceuticals, from water and soil.

## Supplementary Information

Supplementary Information.

## Data Availability

The authors declare that the data supporting the findings of this study are available within the paper and its Supplementary Information files. Should any raw data files be needed in another format they are available from the corresponding author upon reasonable request.

## References

[1] Chai, W. S. et al. A review on conventional and novel materials towards heavy metal adsorption in wastewater treatment application. *J. Clean. Prod.* **296**, 126589 (2021).

[2] Szczepanik, B. Photocatalytic degradation of organic contaminants over clay-TiO2 nanocomposites: A review. *Appl. Clay Sci.* **141**, 227–239 (2017).

[3] Gunatilake, S. K. Methods of removing heavy metals from industrial wastewater. *Methods* **1**, 14 (2015).

[4] Han, H. et al. A critical review of clay-based composites with enhanced adsorption performance for metal and organic pollutants. *J. Hazard. Mater.* **369**, 780–796 (2019). 30851518 10.1016/j.jhazmat.2019.02.003

[5] Burakov, A. E. et al. Adsorption of heavy metals on conventional and nanostructured materials for wastewater treatment purposes: A review. *Ecotoxicol. Environ. Saf.* **148**, 702–712 (2018). 29174989 10.1016/j.ecoenv.2017.11.034

[6] Yu, F. et al. Adsorption behavior of the antibiotic levofloxacin on microplastics in the presence of different heavy metals in an aqueous solution. *Chemosphere* **260**, 127650 (2020). 32693263 10.1016/j.chemosphere.2020.127650

[7] Ajiboye, T. O., Oyewo, O. A. & Onwudiwe, D. C. Simultaneous removal of organics and heavy metals from industrial wastewater: A review. *Chemosphere* **262**, 128379 (2021). 33182079 10.1016/j.chemosphere.2020.128379

[8] Rashed, M. N. & Palanisamy, P. N. Introductory chapter: Adsorption and ion exchange properties of zeolites for treatment of polluted water. *Zeolites Appl.*10.5772/intechopen.77190 (2018).

[9] Gomaa, H. et al. Mesoscopic engineering materials for visual detection and selective removal of copper ions from drinking and waste water sources. *J. Hazard. Mater.* **406**, 124314 (2021). 33168312 10.1016/j.jhazmat.2020.124314

[10] Singh, N. B., Nagpal, G. & Agrawal, S. Water purification by using adsorbents: A review. *Environ. Technol. Innov.* **11**, 187–240 (2018).

[11] Hong, M. et al. Heavy metal adsorption with zeolites: The role of hierarchical pore architecture. *Chem. Eng. J.* **359**, 363–372 (2019).

[12] Wang, B., Lan, J., Bo, C., Gong, B. & Ou, J. Adsorption of heavy metal onto biomass-derived activated carbon. *RSC Adv.* **13**, 4275–4302 (2023). 36760304 10.1039/d2ra07911a PMC9891085

[13] Deng, J. et al. Enhanced sludge solid-liquid separation based on Fe2+/periodate conditioning coupled with polyoxometalates: Cell destruction and protein adsorption. *J. Environ. Manage.* **373**, 123552 (2025). 39632306 10.1016/j.jenvman.2024.123552

[14] Wang, Y. et al. Efficient removal of dibutyl phthalate from aqueous solutions: Recent advances in adsorption and oxidation approaches. *React. Chem. Eng.*10.1039/D4RE00055B (2024).

[15] Li, B. et al. O3 oxidation excited by yellow phosphorus emulsion coupling with red mud absorption for denitration. *J. Hazard. Mater.* **403**, 123971 (2021). 33265012 10.1016/j.jhazmat.2020.123971

[16] Li, B. et al. Simultaneous removal of SO2 and NO using a novel method with red mud as absorbent combined with O3 oxidation. *J. Hazard. Mater.* **392**, 122270 (2020). 32086090 10.1016/j.jhazmat.2020.122270

[17] Xie, Y. et al. Stress-mediated copper-molybdenum alloy enables boosted hydrogen evolution activity. *Acta Mater.* **286**, 120706 (2025).

[18] Ali Alshehri, M. & Pugazhendhi, A. Biochar for wastewater treatment: Addressing contaminants and enhancing sustainability: Challenges and solutions. *J. Hazard. Mater. Adv.* **16**, 100504. 10.1016/j.hazadv.2024.100504 (2024).

[19] Yu, Y. et al. Green recycling of end-of-life photovoltaic modules via Deep-Eutectic solvents. *Chem. Eng. J.* **499**, 155933 (2024).

[20] Unuabonah, E. I., Ugwuja, C. G., Omorogie, M. O., Adewuyi, A. & Oladoja, N. A. Clays for efficient disinfection of bacteria in water. *Appl. Clay Sci.* **151**, 211–223 (2018).

[21] Mukhopadhyay, R. et al. Clay–polymer nanocomposites: Progress and challenges for use in sustainable water treatment. *J. Hazard. Mater.* **383**, 121125 (2020). 31541959 10.1016/j.jhazmat.2019.121125

[22] Kalotra, S. & Mehta, R. Synthesis of polyaniline/clay nanocomposites by in situ polymerization and its application for the removal of Acid Green 25 dye from wastewater. *Polym. Bull.* **78**, 2439–2463 (2021).

[23] Nigam, V. & Lal, G. Review on recent trends in polymer layered clay nanocomposites. *Indian Natn. Sci. Acad. Proc.* **74**, 87–96 (2008).

[24] Benhebal, H., Chaib, M., Leonard, A., Crine, M. & Lambert, S. D. Preparation of polyaniline-modified local clay and study of its sorption capacity. *J. Nanostruct. Chem.* **4**, 1–6 (2014).

[25] Doyo, A. N., Kumar, R. & Barakat, M. A. Facile synthesis of the polyaniline@ waste cellulosic nanocomposite for the efficient decontamination of copper (II) and phenol from wastewater. *Nanomaterials* **13**, 1014 (2023). 36985909 10.3390/nano13061014 PMC10059074

[26] Hajjaoui, H., Soufi, A., Boumya, W., Abdennouri, M. & Barka, N. Polyaniline/nanomaterial composites for the removal of heavy metals by adsorption: A review. *J. Compos. Sci.* **5**, 233 (2021).

[27] Abedi, S. & Abdouss, M. A review of clay-supported Ziegler-Natta catalysts for production of polyolefin/clay nanocomposites through in situ polymerization. *Appl. Catal. A* **475**, 386–409 (2014).

[28] Liu, D., Du, X. & Meng, Y. Facile synthesis of exfoliated polyaniline/vermiculite nanocomposites. *Mater. Lett.* **60**, 1847–1850 (2006).

[29] Vijayakumar, B., Anjana, K. O. & Rao, G. R. 1 edn 012112 (IOP Publishing).

[30] Bekri-Abbes, I. & Srasra, E. Electrical and dielectric properties of polyaniline and polyaniline/montmorillonite nanocomposite prepared by solid reaction using spectroscopy impedance. *J. Nanomater.* **16**, 428–428 (2015).

[31] Tang, Z., Liu, P., Guo, J. & Su, Z. Preparation of polyaniline/vermiculite clay nanocomposites by in situ chemical oxidative grafting polymerization. *Polym. Int.* **58**, 552–556 (2009).

[32] Piri, S. et al. Potential of polyaniline modified clay nanocomposite as a selective decontamination adsorbent for Pb (II) ions from contaminated waters; Kinetics and thermodynamic study. *J. Environ. Health Sci. Eng.* **14**, 1–10 (2016). 27833754 10.1186/s40201-016-0261-z PMC5103387

[33] Mu’azu, N. D., Bukhari, A. & Munef, K. Effect of montmorillonite content in natural Saudi Arabian clay on its adsorptive performance for single aqueous uptake of Cu (II) and Ni (II). *J. King Saud University-Sci.* **32**, 412–422 (2020).

[34] Katsou, E., Malamis, S., Tzanoudaki, M., Haralambous, K. J. & Loizidou, M. Regeneration of natural zeolite polluted by lead and zinc in wastewater treatment systems. *J. Hazard. Mater.* **189**, 773–786 (2011). 21470771 10.1016/j.jhazmat.2010.12.061

[35] Rashed, M. N., Arfien, A. A. & El-Dowy, F. A. Adsorption of heavy metals on chemically modified muscovite. *Aswan University J. Environ. Stud.* **1**, 183–203 (2020).

[36] Bao, T. et al. Catalytic degradation of P-chlorophenol by muscovite-supported nano zero valent iron composite: Synthesis, characterization, and mechanism studies. *Appl. Clay Sci.* **195**, 105735 (2020).

[37] Rashed, M. N., Arifien, A. E. & El-Dowy, F. A. Preparation and characterization of nanomuscovite by intercalation method for adsorption of heavy metals from polluted water. *Environ. Geochem. Health* **5**, 1–18 (2023). 10.1007/s10653-023-01545-4 PMC10310564 37074498

[38] Yuan, J. et al. Green synthesis of nano-muscovite and niter from feldspar through accelerated geomimicking process. *Appl. Clay Sci.* **165**, 71–76 (2018).

[39] Chen, S., Chen, F., Di, Y., Han, S. & Zhu, X. Preparation and characterisation of exfoliated muscovite/poly (2, 3-dimethylaniline) nanocomposites with an enhanced anticorrosive performance. *Micro Nano Lett.* **15**, 509–513 (2020).

[40] Yu, X. The preparation and characterization of cetyltrimethylammonium intercalated muscovite. *Microporous Mesoporous Mater.* **98**, 70–79 (2007).

[41] Ismail, N. H. C., Bakhtiar, N. S. A. A. & Akil, H. M. Effects of cetyltrimethylammonium bromide (CTAB) on the structural characteristic of non-expandable muscovite. *Mater. Chem. Phys.* **196**, 324–332 (2017).

[42] Tetteh, S., Ofori, A., Quashie, A., Jääskeläinen, S. & Suvanto, S. Modification of kaolinite/muscovite clay for the removal of Pb (II) ions from aqueous media. *Phys. Sci. Rev.*10.1515/9783110783643-005 (2022).

[43] Senthilnathan, A., Dissanayake, S., Rathuwadu, N., Mantilaka, P. G. & Rajapakse, R. M. G. Preparation of Muscovite Nanocomposites of Polyaniline with Superior Anti-Corrosive Property as the Protective Coating on Mild Steel Surfaces. *Conference: 36th Annual Technical Sessions of Geological Society of Sri LankaAt: Colombo, Sri Lanka* (2020).

[44] Shabani-Nooshabadi, M., Ghoreishi, S. M., Jafari, Y. & Kashanizadeh, N. Electrodeposition of polyaniline-montmorrilonite nanocomposite coatings on 316L stainless steel for corrosion prevention. *J. Polym. Res.* **21**, 1–10 (2014).

[45] Ding, S. L., Xu, B. H., Liu, Q. F. & Sun, Y. Z. Preparation of nano-kaolinite and mechanism. *Adv. Mater. Res.* **204**, 1217–1220 (2011).

[46] Jokar, M., Mirghaffari, N., Soleimani, M. & Jabbari, M. Preparation and characterization of novel bio ion exchanger from medicinal herb waste (chicory) for the removal of Pb^2+^ and Cd^2+^ from aqueous solutions. *J. Water Process Eng.* **28**, 88–99. 10.1016/j.jwpe.2019.01.007 (2019).

[47] Pillai, S. S. et al. Biosorption of Cd(II) from aqueous solution using xanthated nano banana cellulose: Equilibrium and kinetic studies. *Ecotoxicol. Environ. Saf.* **98**, 352–360. 10.1016/j.ecoenv.2013.09.003 (2013). 24091040 10.1016/j.ecoenv.2013.09.003

[48] Gapusan, R. B. & Balela, M. D. L. Adsorption of anionic methyl orange dye and lead (II) heavy metal ion by polyaniline-kapok fiber nanocomposite. *Mater. Chem. Phys.* **243**, 122682 (2020).

[49] Mahmoud, M. E. & Fekry, N. A. Fabrication of engineered silica-functionalized-polyanilines nanocomposites for water decontamination of cadmium and lead. *J. Polym. Environ.* **26**, 3858–3876 (2018).

[50] Ahmad, R., Hasan, I. & Mittal, A. Adsorption of Cr (VI) and Cd (II) on chitosan grafted polyaniline-OMMT nanocomposite: isotherms, kinetics and thermodynamics studies. *Desalin. Water Treat.* **58**, 144–153 (2017).

[51] Nasrullah, A., Bhat, A. H., Naeem, A., Isa, M. H. & Danish, M. High surface area mesoporous activated carbon-alginate beads for efficient removal of methylene blue. *Int. J. Biol. Macromol.* **107**, 1792–1799 (2018). 29032214 10.1016/j.ijbiomac.2017.10.045

[52] Hua, J. Adsorption of low-concentration arsenic from water by co-modified bentonite with manganese oxides and poly (dimethyldiallylammonium chloride). *J. Environ. Chem. Eng.* **6**, 156–168 (2018).

[53] Rafiei, H. R., Shirvani, M. & Ogunseitan, O. A. Removal of lead from aqueous solutions by a poly (acrylic acid)/bentonite nanocomposite. *Appl. Water Sci.* **6**, 331–338 (2016).

[54] Yadav, V. B., Gadi, R. & Kalra, S. Adsorption of lead on clay-CNT nanocomposite in aqueous media by UV-Vis-spectrophotometer: kinetics and thermodynamic studies. *Emerg. Mater.* **2**, 441–451 (2019).

[55] Tan, Y., Chen, M. & Hao, Y. High efficient removal of Pb (II) by amino-functionalized Fe3O4 magnetic nano-particles. *Chem. Eng. J.* **191**, 104–111 (2012).

[56] Karimi, Z., Khalili, R. & Ali Zazouli, M. Surface modified polythiophene/Al2O3 and polyaniline/Al2O3 nanocomposites using poly (vinyl alcohol) for the removal of heavy metal ions from water: kinetics, thermodynamic and isotherm studies. *Water Sci. Technol.* **84**, 182–199 (2021). 34280163 10.2166/wst.2021.224

[57] Msaadi, R., Ammar, S., Chehimi, M. M. & Yagci, Y. Diazonium-based ion-imprinted polymer/clay nanocomposite for the selective extraction of lead (II) ions in aqueous media. *Eur. Polymer J.* **89**, 367–380 (2017).

[58] Negm, S. H. et al. Appreciatively efficient sorption achievement to U (VI) from the El Sela Area by ZrO2/Chitosan. *Separations* **9**, 311 (2022).

[59] Zhu, S. et al. Magnetic Nanoscale Zerovalent Iron Assisted Biochar: Interfacial Chemical Behaviors and Heavy Metals Remediation Performance. *ACS Sustain. Chem. Eng.* **5**, 9673–9682 (2017).

[60] Reddy, D.H.K. & Lee, S.-M. Magnetic biochar composite: Facile synthesis, characterization, and application for heavy metal removal. Colloids Surfaces A Physicochem. *Eng. Asp.* **454**, 96–103 (2014).

[61] Huda Bonusa Nabila , Endang Tri Wahyuni , Mudasir Mudasir. Simultaneous adsorption of Pb(II) and Cd(II) in the presence of Mg(II) ion using eco-friendly immobilized dithizone on coal bottom ash. *South African J. Chem. Eng*. **45**, 315–327 (2023).

[62] Jiang, M.-Q., Jin, X.-Y., Lu, X.-Q. & Chen, Z.-l. Adsorption of Pb(II), Cd(II), Ni(II) and Cu(II) onto natural kaolinite clay. *Desalination* **252**(1–3), 33–39 (2010).

[63] Shao, D., Chen, C. & Wang, X. Application of polyaniline and multiwalled carbon nanotube magnetic composites for removal of Pb(II). *Chem Eng J.* **185–186**, 144–150 (2012).

[64] Baranchiyeva, Z., Seilkhanova, G., Rakhym, A., Mastai, Y. & Ussipbekova, Y. Adsorption of Pb(II) and Cd(II) from Aqueous Solutions on Polyvinylpyrrolidone Modified Kyzylsok Natural Clay. *Int. J. Biol. Chem.* **14**(1), 164–171 (2021.).

[65] Eftekhari, S., Habibi-Yangjeh, A. & Sohrabnezhad, S. Application of AlMCM-41 for competitive adsorption of methylene blue and rhodamine B: Thermodynamic and kinetic studies. *J. Hazard. Mater.* **178**, 349–355 (2010). 20167425 10.1016/j.jhazmat.2010.01.086

[66] Bany-Aiesh, H., Banat, R. & Al-Sou’od, K. Kinetics and adsorption isotherm of ibuprofen onto grafted [Beta]-CD/chitosan polymer. *Am. J. Appl. Sci.* **12**, 917 (2015).

[67] Srivastava, P. & Hasan, S. H. Biomass of mucor heimalis for the biosorption of cadmium from aqueous solutions: equilibrium and kinetic studies. *BioRes.*10.15376/biores.6.4.3656-3675 (2011).

[68] Dawodu, F. A. & Akpomie, K. G. Kinetic, equilibrium, and thermodynamic studies on the adsorption of cadmium (II) ions using “Aloji Kaolinite” mineral. *Pac. J. Sci. Technol* **15**, 268–276 (2014).

[69] Hosseini-Bandegharaei, A. et al. Sorption efficiency of three novel extractant-impregnated resins containing vesuvin towards Pb (II) ion: effect of nitrate and amine functionalization of resin backbone. *Colloids Surf. Physicochem. Eng. Asp.* **504**, 62–74 (2016).

[70] Ghosal, P. S. & Gupta, A. K. Determination of thermodynamic parameters from Langmuir isotherm constant-revisited. *J. Mol. Liq.* **225**, 137–146 (2017).

[71] Anoja Kawsihan, Sandun Dissanayake, Nadeesha Rathuwadu, Prasanga Gayanath Mantilaka & Rajapakse, R. M. G. in *36th Annual Technical Sessions of Geological Society of Sri Lanka* (Colombo, Sri Lanka, February 2020).

