# Supplementary material for: Muscovite based polyaniline nanocomposite as effective adsorbent for removal of Cd2+ and Pb2+ ions from liquid waste
Source: Sci Rep. 2025 Jun 20;15:20234. doi: 10.1038/s41598-025-99686-2 (PMC12181242; doi:10.1038/s41598-025-99686-2)
Supplement: Supplementary file 1 — Supplementary Information. [file 41598_2025_99686_MOESM1_ESM.pdf]

Table S1: Adsorption Isotherm data for Pb^{2+} adsorption on polyaniline muscovite [PANI/Msc] nanocomposite according to initial metal concentrate variation at 25°C.

Conc. ppm	% of Pb^{2+} adsorbed	C_e	q_e	C_e/q_e	Log C_e	Log q_e	E^2	Ln C_e	Ln q_e	$1/C_e$
10	77.9	2.21	3.895	0.567	0.34	0.59	872607	0.79	1.36	0.452
20	81.2	3.76	8.12	0.463	0.58	0.91	348294	1.32	2.094	0.266
30	89.6	3.12	13.44	0.232	0.49	1.13	484067	1.14	2.598	0.321
50	95	2.5	23.75	0.105	0.4	1.38	709009	0.92	3.168	0.4
75	97	2.25	36.375	0.062	0.35	1.56	846835	0.81	3.594	0.444
100	93.9	6.1	46.95	0.13	0.79	1.67	144323	1.81	3.849	0.164

Table S2 : Adsorption Isotherm data for Cd^{2+}adsorption on polyaniline muscovite [PANI/Msc] nanocomposite according to initial metal concentrate variation at 25°C.

Conc. ppm	% of Cd^{2+} adsorbed	C_e	q_e	C_e/q_e	Log C_e	Log q_e	E^2	Ln C_e	Ln q_e	$1/C_e$
10	76.2	2.38	3.81	0.625	0.377	0.581	770568	0.87	1.34	0.42
20	80.6	3.88	8.06	0.481	0.589	0.906	329306	1.36	2.09	0.26
30	86.8	4	13.02	0.304	0.598	1.115	317499	1.38	2.57	0.25
50	93	3.5	23.25	0.151	0.544	1.366	395538	1.25	3.15	0.29
75	95.2	3.6	35.7	0.101	0.556	1.553	376287	1.28	3.58	0.28
100	92	8	46	0.174	0.903	1.663	86880	2.08	3.83	0.13

Table S3: Kinetic effect of contact time and initial metal concentration (75 ppm) on the adsorption of lead by polyaniline muscovite [PANI/Msc] nanocomposite at 25°C.

Time (t) min	% of removal	C_e	q_t	Log $(q_e - q_t)$	t/q_t	Ln t	$t^{1/2}$
30	89	8.25	33.375	0.559	0.899	3.401	5.48
60	98.6	1.05	36.975	1.6	1.623	4.094	7.75
120	97	2.25	36.375	0.2	3.299	4.787	10.95
240	89	8.25	33.375	0.559	7.191	5.481	15.49

Table S4: Kinetic effect of contact time and initial metal concentration (75 ppm) on the adsorption of cadmium by polyaniline muscovite [PANI/Msc] nanocomposite at 25°.

Time (t) min	% of removal	C_e	q_t	Log $(q_e - q_t)$	t/q_t	Ln t	$t^{1/2}$
30	87.2	9.6	32.7	4.1	0.613	0.92	3.4
60	97.8	1.65	36.675	0.125	-0.903	1.64	4.09
120	95.2	4	35.7	1.1	0.041	3.36	4.79
240	88.1	8.925	33.038	3.7625	0.575	7.26	5.48
